# Correlation of real-time haemoglobin oxygen saturation monitoring during photodynamic therapy with microvascular effects and tissue necrosis in normal rat liver

**DOI:** 10.1038/sj.bjc.6602036

**Published:** 2004-07-20

**Authors:** J H Woodhams, L Kunz, S G Bown, A J MacRobert

**Affiliations:** 1 National Medical Laser Centre, Academic Division of Surgical Specialities, Royal Free and University College Medical School, University College London, Charles Bell House, 67-73 Riding House Street, London W1W 7EJ, UK; 2 Anatomical Institute, University of Munich, Biedersteiner Straße 29, D-80802 München, Germany

**Keywords:** photodynamic therapy, aluminium disulphonated phthalocyanine, haemoglobin oxygen saturation, ischaemia reperfusion injury, reflectance spectroscopy

## Abstract

Photodynamic therapy (PDT) requires a photosensitising drug, light and oxygen. While it is known that the haemoglobin oxygen saturation (HbSat) can be altered by PDT, little has been done to correlate this with microvascular changes and the final biological effect. This report describes such studies on the normal liver of rats sensitised with aluminium disulphonated phthalocyanine. In total, 50 J of light at 670 nm, continuous or fractionated at 25 or 100 mW, was applied with a single laser fibre touching the liver surface. HbSat was monitored continuously 1.5–5.0 mm from the laser fibre using visible light reflectance spectroscopy (VLRS). Vascular shutdown was assessed by fluorescein angiography 2−40 min after light delivery. Necrosis was measured at post mortem 3 days after PDT. In all treatment groups at a 1.5 mm separation, HbSat fell to zero with little recovery after light delivery. At 2.5 mm, HbSat also decreased during light delivery, except with fractionated light, but then recovered. The greatest recovery of fluorescein perfusion after PDT was seen using 25 mW, suggesting an ischaemia/reperfusion injury. Necrosis was more extensive after low power and fractionated light than with 100 mW, continuous illumination. We conclude that VLRS is a useful technique for monitoring HbSat, although the correlation between HbSat, fluorescein exclusion and necrosis varied markedly with the light delivery regimen used.

Photodynamic therapy (PDT) is a treatment for a range of malignant and benign conditions using light-activated photosensitising drugs in the presence of molecular oxygen (Dougherty *et al*, 1998). PDT causes tissue damage by a combination of processes involving the production of reactive oxygen species (in particular singlet oxygen). Studies in experimental tumours suggest that there are three main mechanisms of tissue damage. Firstly, PDT may damage cells directly causing apoptosis and/or necrosis (Luo and Kessel, 1997); secondly, PDT may cause blood flow stasis by vessel constriction, platelet aggregation and/or fibrin plugging. Shortly after light delivery a third process has been noted, where vasoactive lipids, fluid and macromolecules leak from the treated cells, inducing oedema and an inflammatory response (Fingar *et al*, 1992). The response depends on the drug being used, the drug light interval (DLI), the light dose and light power as well as the nature of the tissue being treated and the availability of oxygen (Ochsner, 1997).

Since the cytotoxic effect depends on oxygen, monitoring of tissue oxygenation both during and after PDT is important for understanding the basic physiological mechanisms and dosimetry of PDT (Fingar *et al*, 1992). Both photochemical consumption of oxygen and microvascular shutdown can lead to depletion of molecular oxygen during PDT, limiting the biological effect (Henderson and Fingar, 1987). Photochemical consumption of molecular oxygen can occur through the oxidation of tissue substrate biomolecules by reactive oxygen intermediates generated via Type II and Type I mechanisms (Bonnett, 1999). It has been suggested that tissue oxygenation can be reduced by these processes to levels insufficient for any further PDT effect to occur (Sitnik *et al*, 1998; Henderson *et al*, 2000).

A range of methods is available for monitoring oxygenation changes induced by PDT. Tissue partial pressure of oxygen (pO_2_) has been measured using oxygen microelectrodes to investigate PDT-induced changes *in vivo* with a range of photosensitising agents including porfimer sodium, 5-aminolaevulinic acid-induced protoporphyrin IX (ALA) and benzoporphyrin mono-acid (Curnow *et al*, 2000; Henderson *et al*, 2000; Pogue *et al*, 2001). However, microelectrodes have poor sensitivity at very low levels of pO_2_ and the depth of tissue interrogated has been estimated to be only 15–20 *μ*M (Ince and Sinaasappel, 1999). Electron paramagnetic resonance oximetry has also been used to follow long-term changes in tumour tissue oxygenation in response to benzoporpyhrin mono-acid and ALA PDT (Pogue *et al*, 2002). We have previously used PdTCPP (palladium meso-tetracarboxylphenyl porphine) as a systemically administered oxygen probe and applied PdTCPP phosphorescence lifetime spectroscopy to monitor microvascular changes in oxygen levels in response to PDT with ALA (McIlroy *et al*, 1998).

Noninvasive, optical techniques based on reflectance spectroscopy for the measurement of the oxy- to deoxyhaemoglobin ratio offer an alternative approach. In this study, the application of a new reflectance spectroscopy monitoring system with a restricted wavelength range has been investigated, and is referred to hereafter as the visible light spectrometer (VLRS). The VLRS uses a thin fibre-optic probe that reflectance can be placed at preselected sites on the tissue surface to provide a continuous real-time recording throughout the treatment. This study describes its use for monitoring PDT-induced changes in haemoglobin oxygen saturation (HbSat) in real time and correlating the results with the final biological effect. The importance of vascular effects under the same treatment conditions was assessed using fluorescein angiography.

Fluorescein angiography is an established *in vivo* technique for assessing vascular shutdown and has been used to monitor PDT effects (Bellnier *et al*, 1995). Recovery of perfusion after episodes of temporary ischaemia is known to be a potent instigator of the inflammatory response and is responsible for severe tissue damage in a variety of common conditions such as stroke, myocardial infarction and organ transplant rejection (Zimmerman and Granger, 1994). Ischaemia is associated with the production of xanthine oxidase (XO) while, in parallel, hypoxanthine accumulates because of the breakdown of adenosine triphosphate (ATP) (Zimmerman and Granger, 1994). When oxygen is reintroduced, it enables XO to induce the formation of xanthine from hypoxanthine, resulting in the release of reactive oxygen species, primarily superoxide anions and hydroxyl radicals. There is growing evidence from experiments with other photosensitisers such as porfimer sodium and ALA that reperfusion ischaemia injury may be important (Curnow and Bown, 2002; Korbelik *et al*, 2003).

## MATERIALS AND METHODS

### Animal model

Normal, female Wistar rats (180–220 g, Harlan, Oxon, UK) were used for all experiments. The normal liver was chosen as a suitable model for this study as it is a convenient size in 200 g rats and its homogeneity makes it straightforward to observe and make measurements on the surface during and after PDT, as in our previous dose response studies using phthalocyanine sensitisation (Bown *et al*, 1986). All procedures were performed under general anaesthesia with inhaled Halothane (ICI, Cheshire, UK). Analgesia was administered subcutaneously following surgery (Buprenorphine hydrochloride, Reckitt and Colmann, Hull, UK). All animal experiments were carried out under the authority of project and personal licences granted by the Home Office.

### Photosensitiser

Aluminium disulphonated phthalocyanine (AlS_2_Pc) powder (Prof D Phillips, Imperial College London) was dissolved in physiological strength, phosphate-buffered saline (PBS) at a concentration of 1 mg ml^−1^ and was administered by tail vein injection at a dose of 1 mg kg^−1^ body weight. The disulphonated derivative was chosen for this study since it has been shown to be the most effective for PDT (Chatlani *et al*, 1991).

### Photodynamic therapy studies

Rats were sensitised 1, 3 or 24 h prior to PDT. The liver was exposed at laparotomy, and a 400 *μ*M plane cleaved fibre from a 670 nm Diode laser (Hamamatsu Photonics K.K., Hamamatsu, Japan) was positioned by means of a micromanipulator so that it was just touching the surface of the organ. The key advantages of this method of surface irradiation were firstly that we could position the fibre-optic probe on the surface at precise distances from the laser fibre and, secondly that it produced well-defined lesions, which enabled a quantitative comparison between the monitoring results and PDT-induced damage to be made. Although the light fluence rate where the laser fibre touched the tissue was high, no thermal effect was observed in the light-only control groups. In all cases, the total light dose delivered was 50 J. The illumination regimens investigated were: low power (25 mW) continuous, high power (100 mW) continuous and high power (100 mW) fractionated (5 × 10 J, with 60 s between each fraction). Laser-only and drug-only controls were also carried out. There was a minimum of three animals in each group. All animals were recovered following surgery and killed 3 days later. The minimum (*a*) and maximum (*b*) perpendicular diameters of the nearly circular lesions were measured and the surface area was calculated using the formula *πab*/4. For comparison with the macroscopic findings, representative tissue specimens were fixed in 4% formalin, wax embedded, sectioned and stained with haematoxylin and eosin for examination by light microscopy.

### Monitoring tissue oxygen saturation

**Figure 1:**
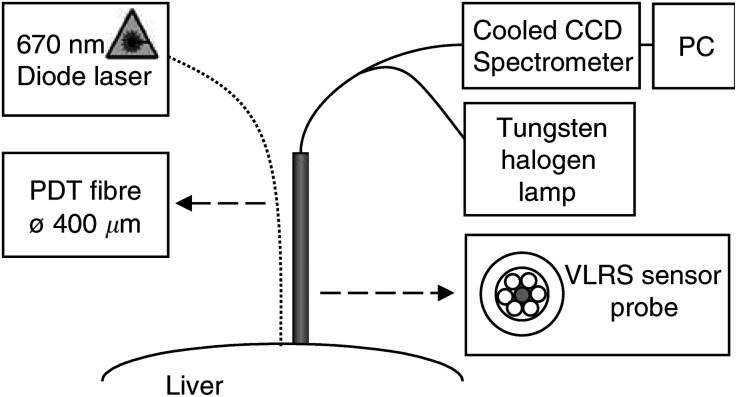
Experimental arrangement for the visible light reflectance spectrometer (VLRS) with the PDT fibre and the VLRS probe positioned on the surface of the liver at a fixed centre-to-centre separation.

Continuous real-time recording of *in vivo* HbSat levels was carried out during and after PDT by means of a PMA-11 spectrograph (Model C7473-36, Hamamatsu Photonics K.K., Hamamatsu, Japan). The PMA-11 is a Czerny-Turner type spectrograph that has a 256 × 128 pixel back-thinned CCD linear image sensor, cooled to −15°C. The system measures simultaneously over a wavelength range of 485–635 nm and a spectral resolution of 0.5 nm that has been calibrated using the bright-line spectrum of a Hg–Ar lamp at 546.07, 576.96 and 579.07 nm. Spectra were collected at 20 Hz, and 10 spectra were averaged to improve the signal to noise ratio. The experimental arrangement of the VLRS system is shown in Figure 1

.

The fibre-optic probe (Avantes BV, Eerbeek, NL) was a bifurcated, hexagonally arranged fibre bundle consisting of seven fibres each with a 200 *μ*M core encased in a 1.5 mm diameter stainless steel tip. The six outer light fibres were coupled to a Tungsten Halogen lamp (Model 77501, Oriel Scientific Ltd, Surrey, UK) that was filtered with a green glass filter (Scott BG 18) and a glass heat-absorbing filter (Schott KG3). The use of the red-absorbing green filter minimised any PDT effect induced by the lamp as confirmed in control studies. The central sensing fibre was coupled to the PMA-11 spectrograph via a standard SMA905 connector. The PMA-11 analysis software (Department of Medical Physics and Bioengineering, University College London, London, UK) calculates the changes in oxyhaemoglobin (HbO) and deoxyhaemoglobin (Hb) in *μ*mol l^−1^ from changes in the spectral attenuation derived from the light reflectance spectra acquired by the spectrometer over its 485–635 nm detection range. From these values, we derived the total haemoglobin (HbT) in *μ*mol l^−1^ as the sum of the HbO and Hb, and the HbSat (expressed as a percentage), which is HbO divided by HbT. The software of the VLRS uses a least-squares fitting algorithm applied over a reduced wavelength range where the wavelength dependence of the differential path length is approximately flat and the depth of penetration into the tissue is approximately constant.

Prior to PDT, the fibre-optic probe was placed on the liver surface at separations of 1.5, 2.5, 3.5 or 5.0 mm from the laser fibre, so that we could monitor tissue sites to which different light doses were delivered. In each case, HbSat was measured for 5 min prior to light delivery to obtain a starting saturation value. Since the VLRS was insensitive to the PDT laser wavelength, we were able to record readings during PDT. The total time of monitoring from the time the laser was switched on was typically 45 min including post-PDT monitoring. There was a minimum of three animals at each separation between the laser fibre and the VLRS probe and in each treatment group.

### Fluorescein angiography

To limit fluorescein leakage into the interstitium, the higher molecular weight derivative, fluorescein isothiocyanate dextran (FITC-Dextran), was used in these studies. This substance is confined to the vasculature, and imaging of the fluorescence allowed us to map which areas are not perfused at various times after light delivery.

Immediately after PDT, 100 mg kg^−1^ bodyweight of FITC-Dextran, (150 000 MW conjugate, Fluka Chemicals Ltd, Dorset, UK) dissolved in physiological strength PBS was injected via the tail vein. The fluorescein was excited using a 3 mW light emitting diode (LED) with peak output at 470 nm (Roithner Lasertechnik, Vienna, Austria) that was directed into a liquid light guide for illumination of the target area. A short focal length lens was connected to the end of the light guide together with a 470±20 nm band pass filter and coupled to a 50/50 C-mounted plate beam splitter (Edmund Optics Ltd, York, UK) so that the excitation beam was perpendicular to the tissue surface. The fluorescence was imaged through the beam splitter mirror at 530 nm (±20 nm) using a band pass filter (Omega Optical Inc., VT, USA) and a long pass filter (Schott OG515) with a sensitive PC-controlled cooled CCD camera (Wright Instruments Ltd, Enfield, UK) capturing a field of 600 × 400 pixels fitted with a video lens capable of × 0.75 magnification (Infinimite video lens, Edmund Optics Ltd, York, UK). Grey scale fluorescence images (<1 s integration time) were captured every 2 min for 40 min after PDT. These images revealed an area of decreased fluorescence centred on the laser fibre. Using Image Toll analysis software (San Antonio, USA), the mean radius of these almost circular zones of fluorescein exclusion was calculated. Control animals received either photosensitiser alone or light alone.

### Statistical analysis

A minimum of three animals was used in each of the PDT and fluorescein angiography groups. Statistical analysis of means in each PDT treatment group was conducted using nonparametric one-way ANOVA. Error bars on all the figures were determined by the standard deviation of the mean.

## RESULTS

### Oxygen saturation measurements

**Figure 2:**
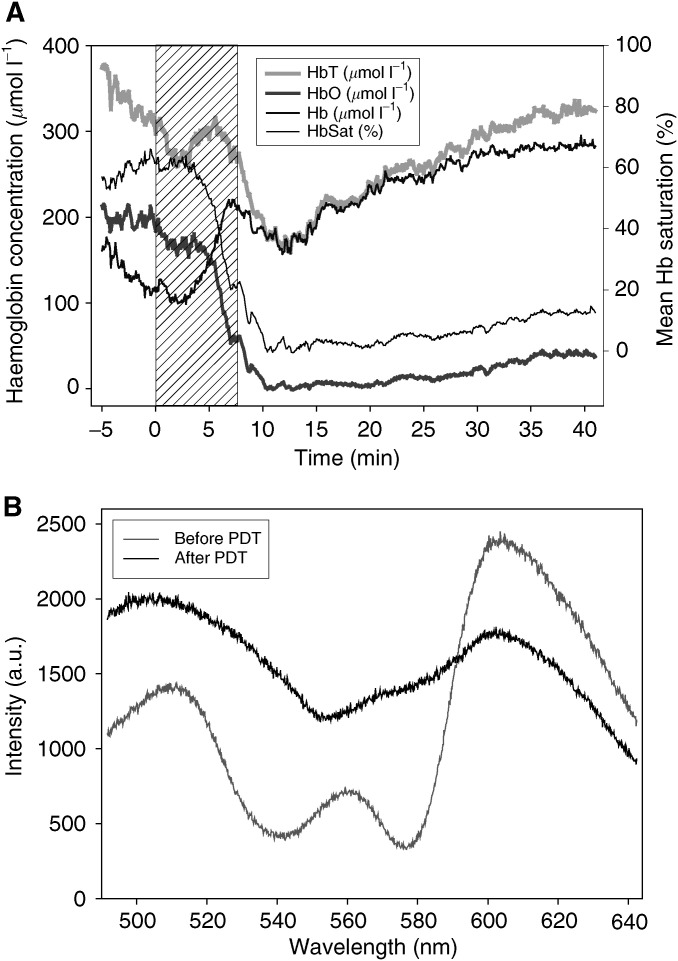
Data collected from a single animal treated (100 mW, 50 J) 24 h after sensitisation with a 1.5 mm fibre to VLRS probe separation showing (**A**) HbT: total haemoglobin. HbO: oxyhaemoglobin. Hb: deoxyhaemoglobin. HbSat (%): percentage haemoglobin oxygen saturation; (**B**) Spectra of remitted intensity before (time 0 min) and after PDT (time 15 min) in the same animal.

**Figure 3:**
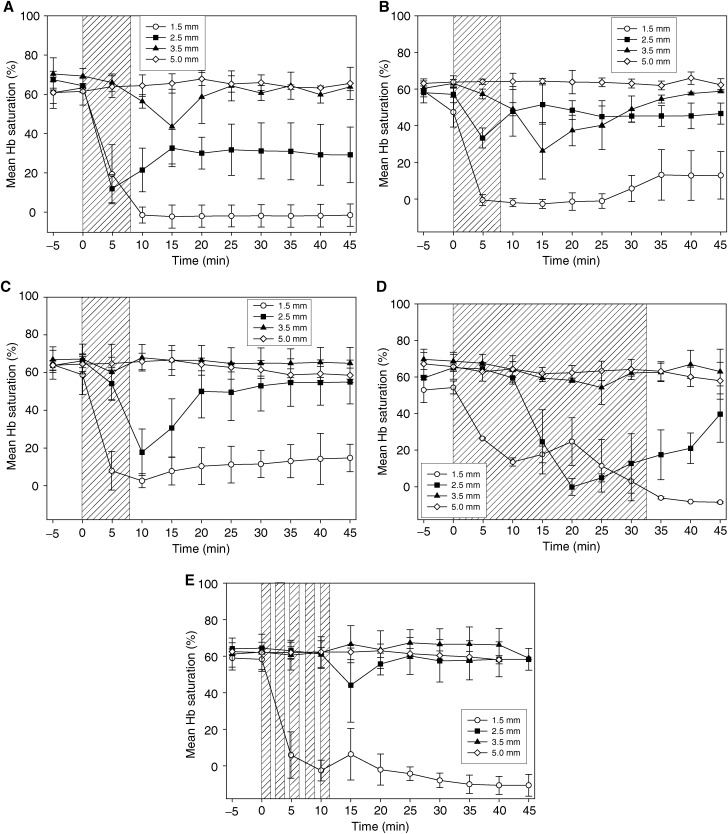
Visible light reflectance spectrometer measurements of HbSat (%) in the normal rat liver before, during and after PDT. Each point represents the mean (with the standard deviation of the mean) from a minimum of three animals. Measurements were made at separations of 1.5, 2.5, 3.5 and 5.0 mm between the VLRS probe and the PDT fibre (one separation per animal). The shaded areas indicate the time when the laser was switched on. All animals were sensitised with 1 mg kg^−1^ body weight AlS_2_Pc and received a total light dose of 50 J at 670 nm. (**A**) High-power continuous treatment (100 mW) with 1 h DLI. (**B**) High-power continuous treatment (100 mW) with 3 h DLI. (**C**) High-power continuous treatment (100 mW) with 24 h DLI. (**D**) Low-power continuous treatment (25 mW) with 24 h DLI. (**E**) High-power treatment with fractionation (100 mW, 5 × 10 J, 60 s between fractions) with 24 h DLI.

The VLRS simultaneously monitors HbO, Hb, HbT and HbSat. Figure 2A

depicts representative data collected from a single animal with the VLRS probe 1.5 mm from the PDT fibre and illustrates the stability of the system over the 45 min monitoring period used. Figure 2B shows spectra of remitted intensity taken from the same animal before PDT (time 0 min) where HbO predominates, and after PDT (time 15 min) where Hb predominates; in the earlier case, the attenuation due to absorption by HbO is clearly evident close to 540 and 580 nm. The main aim of this study was to monitor oxygen saturation, so only the oxygenation results are shown for the other animals. The baseline HbSat in liver prior to PDT was 61±5% (range 49–71%, *n*=40 animals). All animals were treated with a total light dose of 50 J. The effect of varying the light delivery regimen was studied using a drug-light interval of 24 h as our previous work and that of others using AlSPc in normal liver (Bown *et al*, 1986), and small rodent tumours (Tralau *et al*, 1987; Moan and Anholt, 1990) had shown a more homogeneous drug fluorescence distribution at this DLI compared with shorter times. Drug light intervals of 1 and 3 h were studied with a fixed light delivery regimen of 100 mW, continuous. The results are shown in Figure 3A–E

. Control studies with light but without sensitiser showed no changes in HbSat.

In all cases, the HbSat dropped to zero at the 1.5 mm separation between laser fibre and VLRS probe and did not change at the 5.0 mm separation. At the 1.5 mm separation, the reduction was most rapid using high-power (100 mW), continuous illumination. At low power (25 mW), there was a slower rate of decrease, although the drop was just as large by the end of light delivery. In all cases, there was some drop at the 2.5 mm separation except for the fractionated light regimen. The only significant falls in HbSat at 3.5 mm were seen with the short DLIs (1 and 3 h). A summary of the key changes in HbSat at 2.5 and 3.5 mm are shown in Table 1

**Table 1:** Key haemoglobin saturation (HbSat) measurements 2.5 and 3.5 mm from the PDT fibre, and the dimensions of the fluorescein exclusion zones 2 and 40 min after PDT and of the zones of necrosis 3 days after PDT

**Treatment conditions (50 J, 670 nm)**	**DLI (h)**	**% HbSat minimum at 2.5 mm (time from laser on)**	**% HbSat recovery value at 2.5 mm (time from laser on)**	**% HbSat minimum at 3.5 mm (time from laser on)**	**% HbSat recovery value at 3.5 mm (time from laser on)**	**Radius of fluorescein exclusion 2 min post-PDT (mm)**	**Radius of fluorescein exclusion, 40 min post-PDT (mm) (% decrease *vs* 2 min)**	**Maximum radius of necrosis at 3 days (mm)**	**Area of necrosis at 3 days (mm^2^)**
100 mW continuous	1	12 (5 min)	31 (15 min)	42 (15 min)	62 (25 min)	3.4±0.3	3.3±0.4 (3%)	4.0±0.9	39±9
100 mW continuous	3	32 (5 min)	50 (15 min)	25 (15 min)	60 (45 min)	3.3±0.2	2.9±0.3 (12%)	3.9±0.4	37±5
100 mW continuous	24	20 (10 min)	58 (35 min)	60 (5 min)	66 (10 min)	4.3±0.1	3.5±0.2 (19%)	3.6±0.9	38±10
25 mW continuous	24	0 (20 min)	43 (45 min)	57 (25 min)	70 (40 min)	3.5±0.2	2.6±0.2 (26%)	4.9±0.4	60±7
100 mW fractionated (5 × 10 J)	24	46 (15 min)	60 (25 min)	No change	No change	4.1±0.1	3.8±0.1 (7%)	4.6±0.7	53±9

The minimum HbSat values reached and the maximum values attained during recovery are shown, each with the time from switching on the laser at which these were documented. The 40 min post-PDT fluorescein exclusion data includes the percentage reduction compared with the 2 min post-PDT value. Owing to the way the liver lobe was arranged during PDT, the zone of necrosis was elliptical rather than circular. All oxygen measurements were made along the axis where the extent of necrosis was known to be the greatest, so the area of necrosis tabulated here is less than the area corresponding to a circle of the radius documented in the previous column. The zones of fluorescein exclusion were essentially circular.

. The minimum value of HbSat, the recovery value of HbSat post-PDT for each treatment regimen and the time taken to reach those values from when the laser was switched on are shown.

### Fluorescein angiography

**Figure 4:**
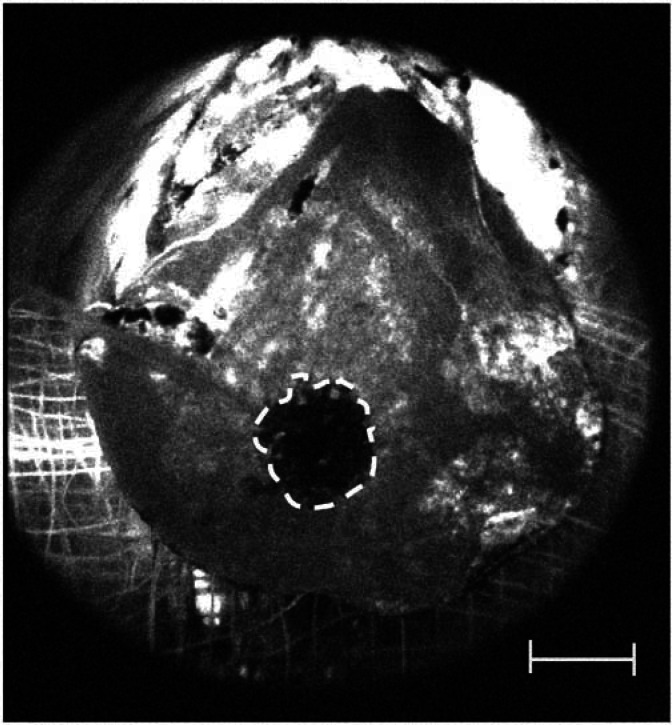
Fluorescein fluorescence image taken 2 min after PDT with a DLI of 3 h and continuous illumination at 100 mW. White scale bar=1 cm. Dashed line shows boundary of fluorescein exclusion zone.

Fluorescein angiography was used to quantify post-PDT changes in microvascular perfusion in the treated portion of the liver, using the same irradiation regimen as in the oxygen-monitoring experiments. In control animals (without light), the green fluorescence was distributed evenly in the liver. In PDT-treated animals, fluorescein was excluded from an area centred on the laser fibre (Figure 4

). The extent and duration of the fluorescein exclusion depended on the treatment parameters. Very little reperfusion was evident with a 1-h DLI, but with a DLI of 24 h and low-power irradiation, reperfusion was documented in almost 50% of the area initially seen to be ischaemic. The mean radius of each fluorescein exclusion zone 2 and 40 min after PDT is shown in Table 1.

### Photodynamic therapy effects

The lesions produced in the liver 3 days after PDT were well defined and easy to measure. Histological examination of representative sections confirmed that macroscopic measurements correlated well with the extent of necrosis seen microscopically. Using continuous irradiation at 25 mW, the surface area of necrosis was approximately 60% larger than using 100 mW and 15% greater than with fractionated irradiation (*P*<0.0001, Student's *t*-test). The results are shown in Table 1.

## DISCUSSION

This study set out to assess a new device for monitoring HbSat during PDT and to look at the possibility of using these oxygenation measurements for predicting the extent of PDT necrosis. Spatially resolved HbSat measurements were carried out using a VLRS system, which proved simple and reliable to use, and provided reproducible measurements in groups of animals treated with a range of different light delivery regimens. The liver HbSat measured by the VLRS in control animals and prior to PDT was remarkably constant at 61±5%, which is similar to the published value of 59±8% measured transcutaneously with near infrared (NIR) spectroscopy in children (Weiss *et al*, 2002). Although NIR spectroscopy can provide the same information as the VLRS technique, the spatial resolution of NIR sensing is poorer owing to the much weaker haemoglobin absorption in the NIR wavelength range. For optical monitoring during PDT, there is also the problem of interference from the PDT laser wavelength to consider. However, when using a 670 nm laser, the scattered light is beyond the VLRS detection range and we were therefore able to record data throughout treatment.

The marked variations in the patterns of HbSat change during and immediately after PDT with different light delivery regimens were unexpected. The only animals in which significant HbSat changes were detected 3.5 mm from the laser fibre were those treated with a short DLI (1 or 3 h). Most likely, these changes were due to vascular effects at a time when the blood level of photosensitiser was high. However, surprisingly, the zone of fluorescein exclusion immediately after light delivery was greater for rats treated with the same light fluence rate (100 mW continuous), but a 24-h drug-light interval (Table 1). Reperfusion was also greater in the 24-h group (correlating well with the recovery in HbSat at 2.5 mm), so the fluorescein exclusion zone 40 min after light delivery was roughly the same in all three, and was comparable to the final area of necrosis.

The other two light delivery regimens applied with a 24-h DLI, that is, low power and fractionated delivery, both produced larger zones of necrosis although different mechanisms may be responsible. The generally accepted rationale for using low-power illumination is that tissue oxygenation levels can be maintained better throughout treatment. With light fractionation, the rationale is that reoxygenation can occur during the dark interval (Foster *et al*, 1991). Low-power illumination has been shown to enhance PDT with several other photosensitisers such as mTHPC (Coutier *et al*, 2002; Tsutsui *et al*, 2002), which was ascribed to lower rates of oxygen consumption. In this study using low power (25 mW), the HbSat at 1.5 mm fell at a slower rate during treatment compared with the 100 mW continuous regimen. At the 2.5 mm separation with low power, the HbSat fell to 0%, but recovered to near pretreatment levels, corresponding to a large reperfusion effect 40 min after PDT, which suggests that under these conditions the vascular effects at and outside the 2.5 mm separation zone were largely reversible (Table 1). Nevertheless, in this case, the final zone of necrosis (radius 4.9 mm) was considerably larger than the 40 min fluorescein exclusion zone (radius 2.6 mm). Reperfusion injury seems likely to have made a much larger contribution here than in the treatments undertaken at 100 mW. The difference between this and the 24 h, 100 mW treatment may be related to the duration of ischaemia, which was more than half an hour here, but less than 10 min in the 100 mW case (Table 1)

In the light fractionation study, there was only a small fall post-treatment in HbSat at a 2.5 mm separation between the fibres (Figure 3E) and only a moderate fluorescein reperfusion effect (Table 1). In this case, we conclude that the larger zone of necrosis compared to continuous irradiation was probably due to the better maintenance of oxygen levels during light delivery. Fractionated irradiation has been shown to improve AlS_4_Pc PDT in a murine tumour model (Anholt and Moan, 1992), although no oxygen measurements were made in that study. Oxygen measurements were made using a microelectrode during PDT with 5-aminolaevulinic acid using continuous or fractionated illumination in the normal rat colon (Curnow *et al*, 2000). Two light fractions were used, 5 and 20 J separated by 150 s, which resulted in a larger area of necrosis compared to continuous irradiation and this effect was ascribed to recovery of oxygen levels during the dark interval.

In summary, using a combination of fluorescein angiography with haemoglobin oxygen saturation monitoring, we have been able to compare noninvasively the responses to PDT under different light delivery regimens. During PDT, vascular shutdown was observed which together with photochemical oxygen consumption resulted in a significant decline in tissue haemoglobin oxygen saturation levels, with the largest drop seen in areas that received the highest light dose. Tissue reperfusion following PDT monitored by fluorescein angiography contributed to the observed reoxygenation. Oxygen back-diffusion from the surrounding nonirradiated tissue and decreased metabolic consumption are assumed to be other factors aiding reoxygenation. Comparing the results of this study with previous work it is clearly apparent, despite the differences in technique and experimental models, that the relationship between tissue oxygenation levels measured during and shortly after light delivery and the final extent of PDT necrosis produced is critically dependent on the treatment conditions. From our observations in normal liver, it would appear that if there is any fall in HbSat, then tissue at that point will be necrosed, but clearly in some cases, as with the low-power illumination (25 mW), the necrosis extends well beyond the point furthest from the laser fibre at which any drop in HbSat is detected. It would be attractive to speculate that if the optimal light delivery conditions (appropriate power and energy level, with or without fractionation) could be predicted from real-time oxygen monitoring, the efficacy of PDT could be improved, although the more data that become available, the more complex the analysis becomes.
